# Molecular Characterization of Fungal Biodiversity in Long-Term Polychlorinated Biphenyl-Contaminated Soils

**DOI:** 10.3390/microorganisms9102051

**Published:** 2021-09-28

**Authors:** Camille Marchal, Joaquim Germain, Muriel Raveton, Blandine Lyonnard, Cindy Arnoldi, Marie-Noëlle Binet, Bello Mouhamadou

**Affiliations:** Laboratoire d’Ecologie Alpine, UMR 5553 CNRS/USMB Université Grenoble Alpes, CEDEX 9, 38058 Grenoble, France; marchal.cam38@gmail.com (C.M.); joaquim.germain@univ-grenoble-alpes.fr (J.G.); muriel.raveton@univ-grenoble-alpes.fr (M.R.); blandine.lyonnard@univ-grenoble-alpes.fr (B.L.); cindy.arnoldi@univ-grenoble-alpes.fr (C.A.); marie-noelle.binet@univ-grenoble-alpes.fr (M.-N.B.)

**Keywords:** PCB-polluted soils, soil physico-chemical properties, fungal diversity, fungal composition

## Abstract

Polychlorinated biphenyls (PCBs) belong to the organic pollutants that are toxic to humans and harmful to environments. Numerous studies dealing with the impact of PCBs on soil microorganisms have focused on bacterial communities. The effects of PCBs on fungal communities in three different PCB-polluted soils from former industrial sites were investigated using high-throughput sequencing of the internal transcribed spacer 1 region. Significant differences in fungal alpha diversity were observed mainly due to soil physico-chemical properties. PCBs only influenced the richness of the fungal communities by increasing it. Fungal composition was rather strongly influenced by both PCBs and soil properties, resulting in different communities associated with each soil. Sixteen Ascomycota species were present in all three soils, including *Stachybotrys chartarum, Fusarium oxysporum, Penicillium canescens, Penicillium chrysogenum,* *Penicillium citrosulfuratum* and *Penicillium brevicompactum,* which are usually found in PCB-polluted soils, and *Fusarium solani, Penicillium canescens, Penicillium citrosulfuratum* and *Penicillium chrysogenum,* which are known PCB degraders. This study demonstrated that PCBs influence the richness and the composition of fungal communities. Their influence, associated with that of soil physico-chemical properties, led to distinct fungal communities, but with sixteen species common to the three soils which could be considered as ubiquitous species in PCB-polluted soils.

## 1. Introduction

Soil pollution is an important source of environmental challenges. The presence of toxic chemical compounds in soil as a result of human industrial, agricultural and transportation activities negatively affects soil microbiota by their acute toxicity [1]. This indirectly influences a wide range of ecosystem processes, such as carbon and nitrogen cycling, organic matter decomposition, plant growth and plant diversity through nutrient availability [2,3,4]. The toxicity of pollutants directly affects soil microorganisms, leading to the disappearance of sensitive species and the development of resistant species able to use these toxic molecules as a nutrient source [5,6].

Among the toxic chemical compounds, polychlorinated biphenyls (PCBs) are a class of organochlorine compounds containing a biphenyl ring that is chlorinated to form 209 different congeners [7]. PCBs were widely used from the 1930s to the 1970s in industries for their non-flammability and electrical resistance properties, and they had many applications, mainly in electrical equipment [8]. Due to their high global production in the past (around 1 million tons) and their high physico-chemical stability, PCBs have been found and remain persistent in the environment [8]. They represent a serious environmental and health problem due to their high adsorption capacity in soils and sediments, their low solubility in water, their accumulation capacity in adipose tissue and their multiple toxicity for animals and humans [9,10,11,12]. For instance, they are immunotoxic and carcinogenic for animals and humans [10].

Several biological strategies, such as anaerobic and aerobic biodegradation by bacterial strains, rhizodegradation, phytodegradation and mycoremediation, described as economical and environmentally friendly, have been developed to treat PCB-polluted soils [8,13,14,15]. Mycoremediation strategies primarily use model fungal species of ligninolytic Basidiomycota due to their interesting biochemical potential, particularly their capacity to produce the nonspecific oxidative exoenzymes involved in the degradation of numerous organic pollutants [7,16,17,18]. Mycoremediation strategies are also based on the use of native fungal species isolated directly from PCB-contaminated soils and generally used in consortium, i.e., a mixture of several strains previously screened for their biodegradation efficiency [19,20,21,22,23]. While the ligninolytic Basidiomycota displayed partial or low PCB degradation rates compared to those expected probably due to their non-telluric origin, their non-adaptation to PCB-polluted soils or their difficulty to grow in soils due to the competition with indigenous microbiota [13,24], native fungal species could be promising [21,23]. However, even if they have the advantage of being naturally adapted to the soil to be decontaminated, their use to treat a specific soil requires a strain isolation step and a screening procedure, making it long and tedious to obtain the most efficient ones in biodegradation processes. Therefore, characterizing species that are ubiquitous in PCB-polluted soils by studying the fungal diversity of these soils could prove to be relevant in mycoremediation strategies both in terms of convenience and efficiency.

Until now, studies on the diversity of microorganisms in PCB-polluted soils have mainly been focused on bacterial communities and have explored either the impact of PCBs or the soil bioremediation on these communities [25,26,27]. Overall, they showed on the one hand decreases in the abundance and the total diversity of microbial communities in PCB-polluted soils and changes in the composition of communities resulting from the increase in the diversity of specific bacterial species involved in the PCB metabolization [25,27]. On the other hand, they indicated changes in the structure and abundance of the microbial community in response to bioremediation [26]. Conversely, in fungi, very few studies have investigated the fungal communities in the PCB-polluted soils. For instance, a study conducted by Ding et al. [28] on the same *Lolium perenne* rhizospheric soil in which different concentrations of PCBs were added, focused on fungal biomass and showed an increase in fungal biomass in the highly PCB-polluted soils [28]. A study conducted by Stella et al. [16] investigated fungal communities in three compartments of the same PCB-polluted soil and focused on the impact of bioaugmentation by two ligninolytic fungi on the resident fungal communities. Both studies were based on mesocosm experiments in which the residual fungal communities of the soils were subjected to stresses linked to PCBs and biological treatments (plants and fungi) and do not provide information on the actual fungal biodiversity of polluted sites. To our knowledge, no study has analyzed the influence of PCBs on fungal communities present in different sites contaminated with different PCB concentrations. However, such a study could allow us to both understand the distribution of fungal communities in PCB-polluted sites with different physico-chemical properties, and characterize ubiquitous species that are able to develop in different PCB-polluted soils and may be of potential interest for use in bioremediation applications.

The aim of this study was to investigate the fungal biodiversity in three soils from different PCB-polluted sites to determine the influence of PCBs on fungal community structure and diversity, which has not been studied so far. We tested the specific hypothesis that fungal communities of these three soils would be different in terms of (i) alpha diversity, with a possible increase in fungal richness in the most polluted soils, and (ii) beta diversity, with important differences in fungal communities across soils, and that PCBs would have an important influence on these communities. We also sought to determine whether it was possible to obtain ubiquitous species able to grow in these three different soils.

## 2. Materials and Methods

### 2.1. Soil Sampling

The studies were carried out on three different PCB-polluted soils. These three studied soils, supplied by SERPOL (SERFIM group), were excavated soils (excavation depth of approximately 50 cm) from three different old storage sites for electrical materials containing PCBs in France.

Soil C was sampled in Summer 2015, while soil T and soil D were sampled in Summer 2019. Each soil sample was sieved (<4 mm) and homogenized by mixing several times. Six subsamples of each soil (5 kg) were randomly taken and stored at −20 °C. Before chemical and molecular analyses, each soil subsample was defrosted at room temperature and divided into two parts: one part for molecular analyses and one part for chemical analyses.

### 2.2. Soils’ Chemical Properties

Four subsamples (7 g) of each soil were oven-dried at 40 °C for 48 h. Soil of each subsample (5 g) was subsequently oven-dried at 550 °C for 4 h to determine soil organic matter [29]. The last 2 g of soil of each subsample were ground to powder to measure total C and N contents using a FlashEA 1112 elemental analyzer (Fisher Scientific Inc., Waltham, MA, USA) and to determine soil pH in a 1:2.5 (soil:distilled water) solution [30].

Analyses of the 7 indicator PCBs (PCB 28 (2,4,4′ Trichlorobiphenyl), PCB 52 (2,2′,5,5′-Tetrachlorobiphenyl), PCB 101 (2,2′,4,5,5′-Pentachlorobiphenyl), PCB 118 (2′,3,4,4′,5′-Pentachlorobiphenyl), PCB 138 (2,2′,3,4,4′,5′-Hexachlorobiphenyl), PCB 153 (2,2′,4,4′,5,5′-Hexachlorobiphenyl) and PCB 180 (2,2′,3,4,4′,5,5′-Heptachlorobiphenyl)) were carried out on 100 g of each subsample. PCBs were extracted using hexane/acetone (50/50; *v*/*v*) and analyzed by gas chromatography-mass spectrometry by Eurofins Scientific (Saverne, France) according to the standard NF EN 16167 [31]. The soil characteristics are shown in Table 1.

### 2.3. DNA Extraction, PCR Amplification and Illumina-Based Sequencing

For each of the 3 PCB-polluted soils, 6 subsamples were taken, and DNA was extracted from 150 mg of each subsample using the FastDNA^®^ SPIN Kit for Soil (MP Biomedicals, Irvine, CA, USA). After extraction, the DNA was qualitatively and quantitatively tested with a spectrophotometer (Nanodrop, PeqLab, Erlangen, Germany).

The nuclear ribosomal ITS1 was used as a molecular marker for fungal community structure and amplified with the fungal primers ITS5 [32] and 5.8S_fungi [33]. Both primers were extended by sample-specific tags of 8 nt length to allow parallel sequencing of multiple samples. The PCR reactions were carried out as reported [34]. Four PCR reactions (technical replicates) were carried out from DNA extracted from each sample, and the four PCR products from each sample were pooled. PCR products were purified with the QIAquick kit in accordance with the manufacturer’s instructions (Qiagen, Courtaboeuf, France) and DNA was quantified using the Bioanalyzer (Agilent Technologies, Inc., Santa Clara, CA, USA). The purified amplicons were pooled for the sequencing by using equivalent molarities amongst amplicons. The library construction and sequencing (Illumina MiSeq 150 bp pair-end) were carried out by Fasteris (Geneve, Switzerland).

### 2.4. Bioinformatic Analysis

Reads’ assembly and primary filtering were performed using the OBItools package [35]. Short (<100 nt) or rare (occurrence < 2) reads were removed. The ecotag function of OBItools was used to taxonomically assign the obtained unique sequences (identical repeated sequences) to the r143 EMBL fungi database using the ITS5 and 5.8S_fungi primers. Unique sequences belonging to the fungal Kingdom and having a best identity of at least 0.8 were selected, resulting in 1138 unique sequences for 2,647,492 reads in total. Highly similar unique sequences were clustered into Operational Taxonomic Units (OTUs) by computing pairwise similarities with Sumatra (OBItools package, https://metabarcoding.org/sumatra (accessed on 16 May 2016) and forming 98% similarity clusters with the Markov Cluster algorithm (MCL) classification process [36].

The R software 4.0.5 was then used to aggregate unique sequences belonging to the same OTUs. From these sequences, we removed sequences of rare OTUs (found in only one sample) and sequences from samples that have a Bray–Curtis scatter distance which exceeds 95% of their confidence interval. The dataset was then normalized by sample using the Hellinger standardization method (“descostand” function, package vegan). The final contingency table displays the relative abundance (normalized read counts) of 639 OTUs among 18 samples.

### 2.5. Phylogenetic Analysis

Phylogenetic analyses were carried out using the neighbor-joining method [37], based on Clustal W alignments, and the robustness of tree topologies was evaluated by performing bootstrap analysis of 10,000 datasets using MEGA 3.1 [38].

### 2.6. Statistical Analysis

Normality of the data was tested by the Shapiro–Wilk test. The alpha diversity was analyzed using Levene’s test (for the homogeneity of variance) and ANOVA followed by the Tukey test. The *imputePCA* function (package missMDA) was used to impute the missing physico-chemical entries using Correspondence Analysis (CA). The variance partitioning of alpha diversity was measured using redundancy analysis (*varpart, rda* functions). The distribution of fungal communities was evaluated by Principal Component Analysis (PCA). The variance partitioning of beta diversity was measured using PERMANOVA on Bray–Curtis distance matrices (*adonis2* function), followed by the Tukey Honest Significant Differences test. The abundance of OTUs found in the 3 studied soils was compared in pairs using the Chi-squared test and adjusted standardized residuals [39]. All statistical analyses were performed using R package vegan [40].

## 3. Results

### 3.1. Alpha Diversity of Fungal Communities

**Figure 1:**
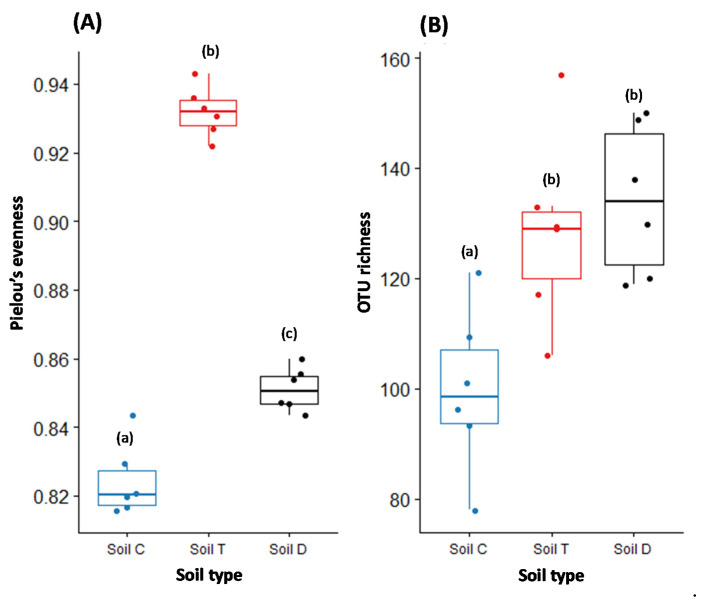
Box plots showing Pielou’s evenness (**A**) and the OTU richness (**B**) of fungal communities. The lower and upper boundaries of each box enclose 25% ± 75% of the data. The line within the box shows the median value, and the bar lines above and below the boxes indicate minimum and maximum values (*n* = 6). Points display raw data. Different small letters, shown in parentheses, indicate significant differences between values (ANOVA followed by Tukey test; *p*-value < 0.05).

First, rarefaction analysis performed on the data of each studied soil indicated that the taxon accumulation curves reached saturation (Supplementary Figure S1). The evenness of fungal communities showed the same pattern and varied significantly depending on the studied soils. It was significantly lower in the least polluted soil C than in soils D and T (Figure 1A). Among these two soils, soil T exhibited the highest evenness of the fungal communities. Regarding the richness, while it was significantly lower in soil C, it did not significantly vary when comparing soils T and D (Figure 1B).

Soil physico-chemical properties (N, C, pH and OM) had a significant effect on the evenness and the richness of fungal communities, while the PCB concentrations had an impact only on the richness of fungal communities (Supplementary Table S1).

### 3.2. Patterns of Soil Fungal Communities

**Figure 2:**
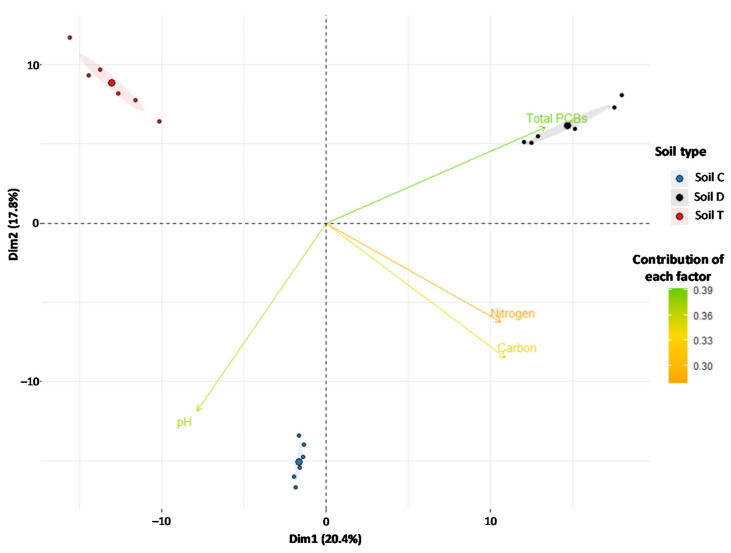
Principal component analysis (PCA) of fungal communities in the studied soils. The analyses were based on the sequences of OTUs present in the three soils taking into account the concentrations of the 7 indicator PCBs as well as the soil physico-chemical factors. The blue, black and red dots correspond respectively to the fungal communities of soils C, D and T. Ellipses represent 95% normal probability.

The study of fungal beta diversity performed by PCA evidenced notable differences in fungal communities across soils. The fungal communities of each soil were clustered (Figure 2). This pattern was confirmed by the variance partitioning of the entire dataset (Supplementary Table S2), which indicated that the total PCB concentrations in soils were the most significant factor determining the fungal beta diversity (explaining 37% of the variance). The physico-chemical properties N, C and pH each had a significant but less pronounced effect, explaining 29%, 11% and 3% of the variance respectively, while OM did not show a significant effect on fungal beta diversity (Supplementary Table S2).

### 3.3. Composition of Soils’ Fungal Communities

**Figure 3:**
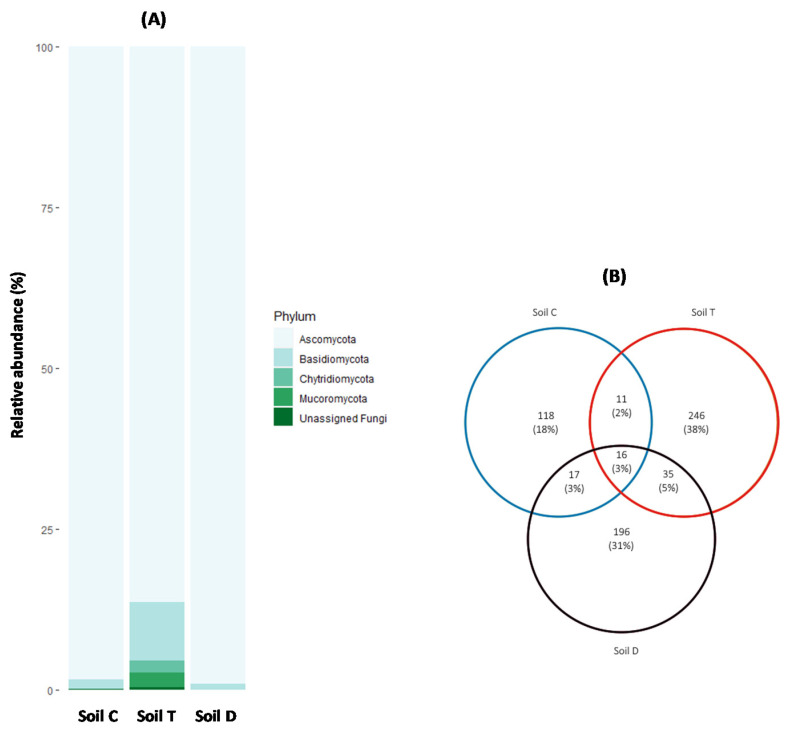
Comparison of fungal communities of the studied soils at phylum (**A**) and OTU (**B**) levels.

The global composition of fungal communities (Figure 3A) evidenced a dominance of Ascomycota in abundance (determined by the number of OTUs) of at least 86% in the three studied soils. Basidiomycota were less abundant, varying between 0.88% and 9%. Chytridiomycota and Mucoromycota were only represented in soil T in low proportions, 1.91% and 2.22%, respectively. Additionally, a low proportion of unclassified fungi was found in soils C (0.13%) and T (0.37%).

Venn analysis (Figure 3B) based on all OTUs present in each polluted soil was performed to identify the OTUs shared by the three studied soils. The largest number of common OTUs shared by two different soils (51 OTUs) was observed between soils T and D exhibiting the highest PCB concentrations (>10 mg kg^−1^). Soils C and T shared 27 common OTUs while soils C and D shared 33 common OTUs. Among all OTUs, only 16 were present in the three studied soils (Figure 3A).

**Figure 4:**
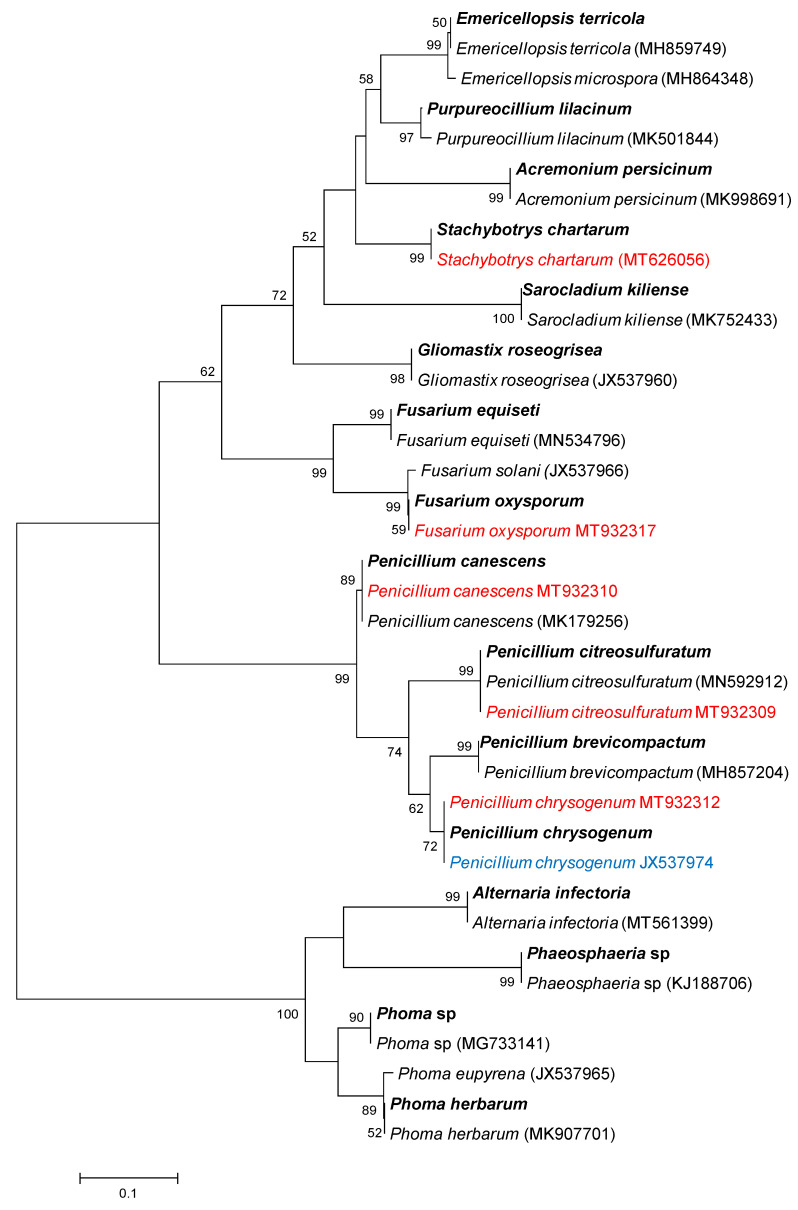
Phylogenetic analyses performed with the neighbor-joining method based on ITS1 sequences. The GenBank accession number of the sequences of fungal species used as reference species are indicated in brackets. The species represented in red and blue are those previously isolated from soil D [22] and from another PCB-polluted soil [20], respectively. The 16 species present in the 3 studied soils are in bold. Bootstrap values exceeding 50% are shown on the branches.

Phylogenetic analysis (Figure 4) was performed to better identify the common OTUs shared by the three PCB-polluted soils. Their sequences were also deposited in the GenBank databases (MZ558754–MZ558769). We included in the analysis the reference sequences available in the GenBank databases to confirm the taxonomic assignments and validate the specific delimitation of each OTU. The 16 species were divided into 12 genera of Ascomycota. Each genus was represented by one species, except the genera *Penicillium* by four species, *Fusarium* by two species and *Phoma* by two species.

**Figure 5:**
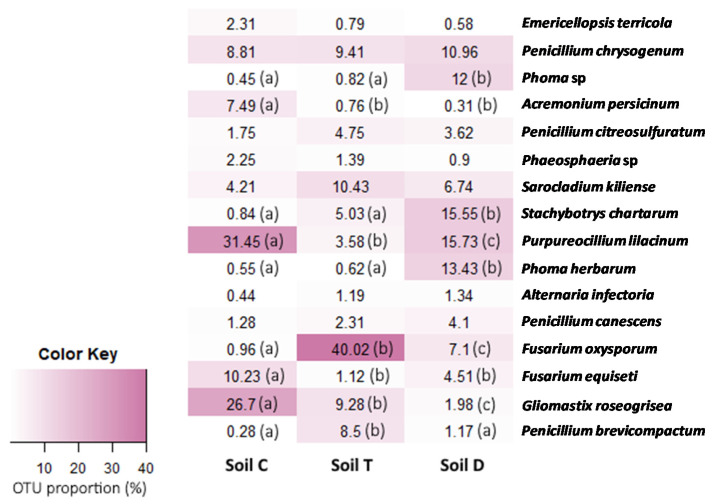
Composition of the common OTUs shared by the three PCB-polluted soils. The proportion of the relative abundance of each OTU was compared two-by-two between the three studied soils using a Chi-squared test (*p*-value < 0.05). Different small letters, shown in parentheses, indicate significant differences between values for each OTU.

The abundances of these 16 OTUs compared to each other were variable depending on the studied soil (Figure 5). Three OTUs were the most abundantly represented, among them, *Purpureocillium lilacinum* and *Gliomastix roseogrisea* were the most abundant in soil C while *Fusarium oxysporum* was the most abundant in soil T. The other OTUs were less represented and most of them were in significantly different proportions in the three soils.

## 4. Discussion

While many studies have focused on the screening of fungal species to characterize the most effective ones in PCB bioremediation, to our knowledge, no study has investigated the influence of PCBs on the structure and composition of fungal communities, which is crucial to identify a set of ubiquitous species that can be used with convenience and efficiency to remediate different PCB-polluted soils. This study was undertaken to fill this gap by investigating the response of fungal communities toward PCB contamination across three long-term polluted soils.

A strong difference in fungal alpha diversity was observed between the three PCB-polluted soils, consistent with the results obtained with other kinds of pollutants [41]. Soil physico-chemical properties affected the evenness and the richness of fungal communities, as demonstrated in the literature [42]. PCB pollution only affected the richness of fungal communities by increasing it, probably by promoting the installation of species able to metabolize these pollutants, as demonstrated in bacterial communities [28]. Overall, no linear relationship between PCB concentrations and fungal alpha biodiversity was found as the highest fungal alpha diversity was observed at intermediate levels of PCBs, thus confirming the great influence of the soil physico-chemical properties on fungal alpha diversity.

Fungal beta diversity was rather strongly driven by both PCB concentrations and the soil physico-chemical properties. While the effects of soil properties on fungal beta diversity are well-documented [43,44,45,46], the effects of PCBs, still unknown, are consistent with their strong impact on fungal richness. These results support the idea that PCBs could select species that, thanks to adaptation systems that are described as constitutive or rapidly inducible by these pollutants [47], were able to tolerate or metabolize them. Such suggestions concerning other organic pollutants have already been reported [41]. These joint effects were reflected in the presence of specific fungal groups associated preferentially with each studied soil. While Ascomycota were dominant in the three PCB-polluted soils, consistent with their well-known resistance to stress and their potentiality to readily use nutrient resources [34,48,49], Basidiomycota were also present in the three studied soils but were very poorly represented (0.88% to 9%). This low representation of Basidiomycota is surprising, as this phylum represents about 30% of described fungal species [50]. This could be due to their ecological traits as mycorrhizal or filamentous wood-decaying species [13,50] or could suggest a lack of adaptation to PCB-polluted soils, and could probably explain their unsatisfactory performance in the PCB mycoremediation processes through bioaugmentation despite their remarkable metabolic properties [13].

The Venn analyses carried out at the OTU level confirmed the results of the beta diversity and showed that among the fungal species characterized, only 16 species were present in varying proportion in the 3 soils.

First, this result suggests that these species were stable and tolerant to environmental stress. Interestingly, among these species, *Stachybotrys chartarum, F. oxysporum, Penicillium canescens, P. chrysogenum* and *P. citrosulfuratum* had previously been isolated from soil D, while *P. chrysogenum*, *P. brevicompactum* and *F. oxysporum* had already been isolated from other soils highly polluted with PCBs [19,20,22], suggesting their dominance and ubiquity in PCB-polluted soils. The fact that the abundance of species was variable according to the soils could confirm the regulation of PCBs and/or physico-chemical properties on these species, consistent with the studies showing the effect of PCBs on the fungal biomass [28].

Second, among the 16 species, the strains of *F. solani, P. canescens, P. citrosulfuratum* and *P. chrysogenum* had already shown interesting abilities for the PCB degradation in liquid medium by displaying biodegradation rates of at least 60% after 7 days of incubation [20,22]. More interestingly, some strains of these species used in consortium in the mycoremediation by bioaugmentation have made it possible to obtain remarkable biodegradation rates using soils highly polluted with PCBs [21,23]. These results suggest that it might be possible to use strains of these 16 species in consortium to potentially effectively remediate PCB-contaminated soils without having to isolate and screen the strains beforehand, without long and tedious steps and without decreasing the efficiency of biodegradation due to a possible lack of growth of bioaugmented strains.

## 5. Conclusions

This original study, focusing on the biodiversity of fungal communities in three PCB-polluted soils, showed that PCBs had a low overall influence on the fungal alpha diversity by only increasing the richness, in contrast to the soil physico-chemical properties which affected the relative abundance, the evenness and the richness of fungal communities. On the other hand, together with the soil physico-chemical properties, PCBs had a significant influence on the composition of fungal communities. The influences of both drivers made it possible to characterize 16 species that could be considered as ubiquitous in PCB-polluted soils.

## Figures and Tables

**Table 1:** Physico-chemical characteristics of PCB-polluted soils. The results are expressed as means ± standard deviation (*n* = 4). The concentrations of each of the 7 PCB congeners and the total concentrations of all the 7 congeners are indicated.

Samples	Soil Properties				Soil PCB Concentrations							
	pH	Organic Matter (g kg^−1^)	Nitrogen (g kg^−1^)	Carbon (g kg^−1^)	PCB28 (mg kg^−1^)	PCB52 (mg kg^−1^)	PCB101 (mg kg^−1^)	PCB118 (mg kg^−1^)	PCB138 (mg kg^−1^)	PCB153 (mg kg^−1^)	PCB180 (mg kg^−1^)	Total PCB (mg kg^−1^)
**Soil C**	9.16 ± 0.02	42.25 ± 1.00	109.75 ± 5.09	57.08 ± 0.77	0.03 ± 0.01	0.22 ± 0.04	0.24 ± 0.04	0.13 ± 0.03	0.45 ± 0.16	0.48 ± 0.13	0.28 ± 0.10	1.81 ± 0.47
**Soil T**	8.70 ± 0.03	34.58 ± 0.34	48.08 ± 14.02	38.65 ± 2.78	0.05 ± 0.00	0.22 ± 0.02	0.78 ± 0.19	0.41 ± 0.05	4.57 ± 1.32	5.16 ± 1.42	4.93 ± 0.28	15.98 ± 2.02
**Soil D**	8.32 ± 0.10	36.18 ± 0.94	117.48 ± 3.94	56.80 ± 1.76	1.32 ± 0.57	37.73 ± 4.80	148.50 ± 13.34	69.28 ± 10.45	222.75 ± 20.25	226.25 ± 17.75	134.78 ± 14.62	843.50 ± 66.78

## Data Availability

Not applicable.

## Supplementary Materials

The following are available online at https://www.mdpi.com/article/10.3390/microorganisms9102051/s1, Figure S1: Rarefaction curves from the number of sequences from the three studied soil samples, Table S1: Effects of soil type and PCB concentrations on the evenness and richness of fungal communities (*** *p* < 0.001, ** *p* < 0.01, * *p* < 0.05), Table S2: Variance partitioning indicating the effects of PCBs and soil physico-chemical properties on the distribution of fungal communities in the 3 studied soils (*** *p* < 0.001, ** *p* < 0.01, * *p* < 0.05).
