# Supplementary material for: Molecular Characterization of Fungal Biodiversity in Long-Term Polychlorinated Biphenyl-Contaminated Soils
Source: Microorganisms. 2021 Sep 28;9(10):2051. doi: 10.3390/microorganisms9102051 (PMC8541467; doi:10.3390/microorganisms9102051)
Supplement: Supplementary file 1 [file microorganisms-09-02051-s001.zip › microorganisms-1380833-supplementary.pdf]

Supplementary materials

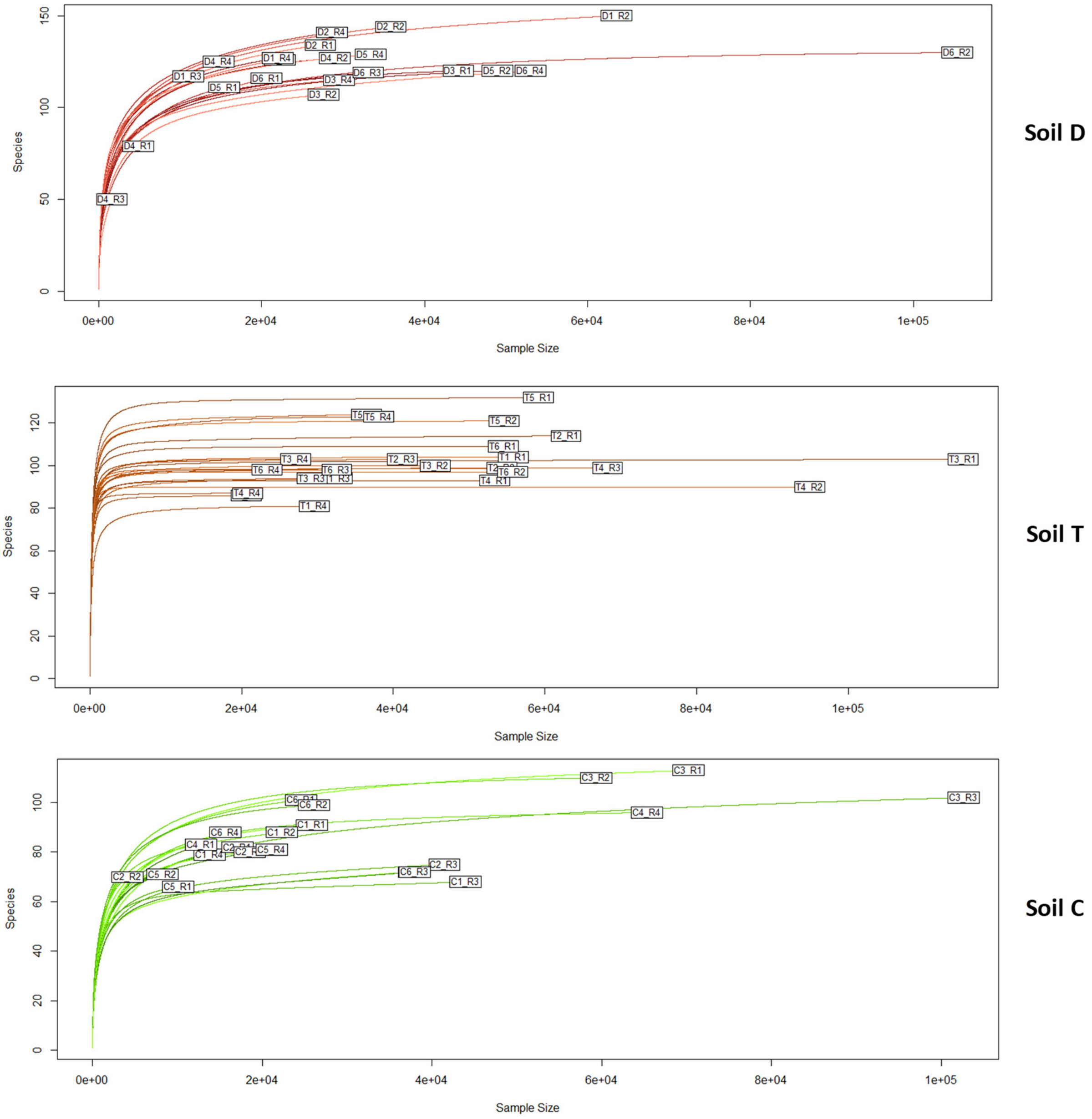

Figure S1. Rarefaction curves from the number of sequences from the three studied soil samples.

Table S1. Effects of soil type and PCB concentrations on the evenness and richness of fungal communities (***$P < 0.001$, **$P < 0.01$, *$P < 0.05$).

			(Evenness)			
	Df	R^2	Adjusted R^2	Variance	F	Pr(>F)
PCB	1	0.0669	0.00858	0.00015255	1.1471	0.295
Soil	4	0.95015	0.93481	0.00216664	61.945	0.001
Residual			0.05685			
Total	18			0.0022803		
			**(Richness)**			
	Df	R^2	Adjusted R^2	Variance	F	Pr(>F)
PCB	1	0.25282	0.20612	113.18	5.4138	0.026
Soil	4	0.62168	0.50527	278.31	5.3405	0.011
Residual			0.53592			

Table S2. Variance partitioning indicating the effects of PCBs and soil physico-chemical properties on the distribution of fungal communities in the 3 studied soils (***$P < 0.001$, **$P < 0.01$, *$P < 0.05$).

	**Df**	**Sum Of Squares**	**R^2**	**F**	**Pr(>F)**
Total PCB	1	2.0671	0.37771	26.3015	0.001
Nitrogen	1	1.5963	0.29168	20.3109	0.001
Carbon	1	0.6155	0.11247	7.8318	0.001
pH	1	0.1921	0.03511	2.4446	0.032
Organic Matter	1	0.0586	0.0107	0.7453	0.535
Residual	12	0.9431	0.17233		
Total	17	5.4727	1		
